# High adaptability of the omega loop underlies the substrate-spectrum-extension evolution of a class A β-lactamase, PenL

**DOI:** 10.1038/srep36527

**Published:** 2016-11-09

**Authors:** Hyojeong Yi, Jin Myung Choi, Junghyun Hwang, Fabio Prati, Thinh-Phat Cao, Sung Haeng Lee, Heenam Stanley Kim

**Affiliations:** 1 Department of Biomedical Sciences, Korea University, Anam-Dong, Seongbuk-Gu, Seoul 136-705, Korea; 2 Department of Cellular and Molecular Medicine, Chosun University School of Medicine, Gwangju 501-759, Korea; 3 Department of Life Sciences, University of Modena, Modena 41125, Italy

## Abstract

The omega loop in β-lactamases plays a pivotal role in substrate recognition and catalysis, and some mutations in this loop affect the adaptability of the enzymes to new antibiotics. Various mutations, including substitutions, deletions, and intragenic duplications resulting in tandem repeats (TRs), have been associated with β-lactamase substrate spectrum extension. TRs are unique among the mutations as they cause severe structural perturbations in the enzymes. We explored the process by which TRs are accommodated in order to test the adaptability of the omega loop. Structures of the mutant enzymes showed that the extra amino acid residues in the omega loop were freed outward from the enzyme, thereby maintaining the overall enzyme integrity. This structural adjustment was accompanied by disruptions of the internal α-helix and hydrogen bonds that originally maintained the conformation of the omega loop and the active site. Consequently, the mutant enzymes had a relaxed binding cavity, allowing for access of new substrates, which regrouped upon substrate binding in an induced-fit manner for subsequent hydrolytic reactions. Together, the data demonstrate that the design of the binding cavity, including the omega loop with its enormous adaptive capacity, is the foundation of the continuous evolution of β-lactamases against new drugs.

A major cause of global-scale antibiotic resistance is the hydrolytic inactivation of β-lactams, the most widely used group of antibiotics, by β-lactamases^1^. This problem prompted researchers to develop new antibiotic variants, such as the extended-spectrum cephalosporin. However, the effectiveness of these antibiotics was challenged by the emergence of the extended-spectrum β-lactamases (ESBLs) that evolved under the selective pressure exerted by these antibiotics^1^,^2^,^3^.

The evolution of the ESBLs has been driven by various mutations, principally those targeting the residues constituting the active-site region^3^,^4^. In class A β-lactamases, a conserved structural element called the omega loop^5^ constitutes a wall at the active site that shapes the binding cavity^6^,^7^,^8^,^9^. This is a prominent site for mutations to occur, resulting in substrate spectrum extension^2^,^4^,^10^,^11^,^12^,^13^,^14^,^15^,^16^,^17^. Most mutations identified in the omega loops of ESBLs were point mutations that resulted in amino acid substitutions^2^,^3^,^4^,^16^. Structural alterations in the omega loop that resulted from amino acid-substitution mutations have been widely studied in association with substrate spectrum extension^18^,^19^. However, other types of mutations, including deletions^20^,^21^ and duplications resulting in tandem repeats (TRs)^10^,^22^, have also been identified. Due to their large sizes, TRs are expected to cause more severe perturbations in omega-loop structures than do amino acid-substitution mutations. Therefore, TRs provide a unique opportunity to explore the adaptive capacity of the omega loop against structural stresses and the resulting molecular consequences as they relate to substrate spectrum extension.

In this study, we demonstrate the extensive perturbation-absorbing capacity of the omega loop against TR mutations of various sizes, some of which are large enough to include the entire omega loop. Then, we describe the structural basis of the mechanism underlying the highly adaptive capacity of the omega loop, which leads to the evolution of β-lactamases that can hydrolyze new drugs by providing a flexible active site.

## Results and Discussion

### TR mutations in the omega loop-coding region in the *penL* gene

We obtained TR mutations of *penL* (*penA* in our previous reports^4^,^10^,^20^,^21^ is renamed *penL* here, following the nomenclature guidelines by Poirel *et al.*^23^), which codes for a class A β-lactamase, by exposing *Burkholderia thailandensis* strain E264^24^ to ceftazidime (3–5 μg/ml), a third-generation cephalosporin. PenL (NCBI reference sequence: YP_439646.1) is highly conserved in pathogenic *Burkholderia* species, including *Burkholderia pseudomallei*, *Burkholderia mallei*, and *Burkholderia cenocepacia*^25^. The antibiotic regimen used to treat infections by these *Burkholderia* pathogens generally includes ceftazidime^26^. PenL has been used as a model in exploring various evolutionary paths to substrate spectrum extension through amino acid substitutions, deletions, and duplications^4^,^10^,^20^,^21^.

**Figure 1:**
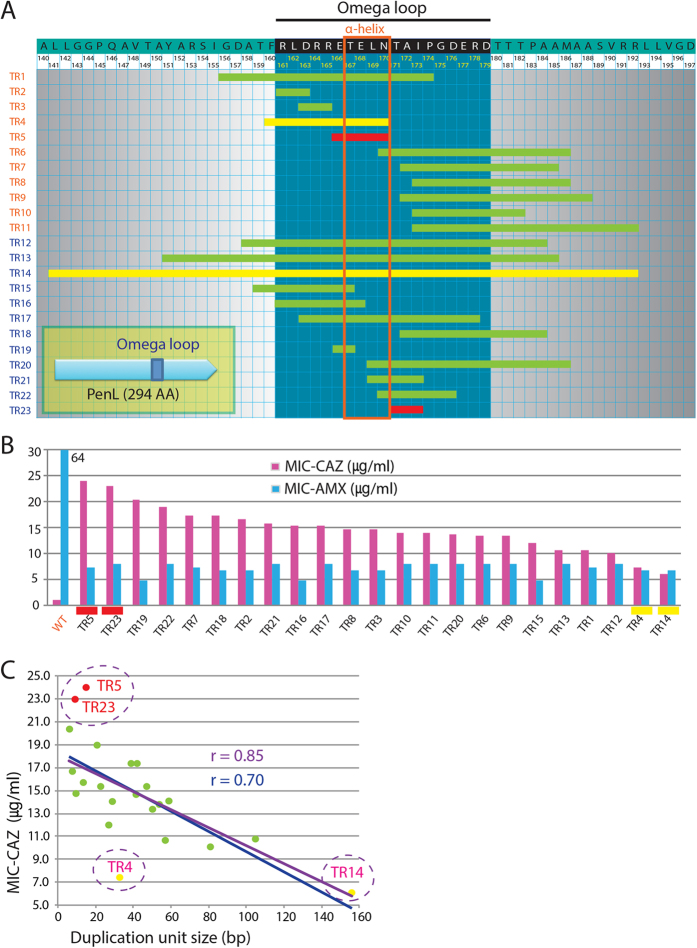
Tandem repeat (TR) mutations conferring ceftazidime resistance in the β-lactamase PenL. (**A**) Duplicated regions in 23 mutants of PenL. The amino acid sequence of wild-type PenL is shown at the top, and the positions are numbered following the Ambler scheme^42^. Duplicated regions in the mutants are shown with horizontal bars colored red (two highest MICs for ceftazidime), yellow (two lowest MICs for ceftazidime), or green (others). The mutants with TRs 1 to 11 have been reported^10^ and are included for comparison. (**B**) The MICs for ceftazidime and amoxicillin of the mutants. The *B. thailandensis* strain E264 with the intact *penL* allele (i.e., *penL*-WT) and other strains with various chromosomal *penL* alleles with TRs, arranged in descending order of MIC for ceftazidime (MIC-CAZ), are shown along with their MICs for amoxicillin (MIC-AMX). The strains with high or low MIC-CAZ values, the duplicated regions of which are color-coded red and yellow in panel A, respectively, are denoted with the same color codes. (**C**) Scatter plot of duplication unit sizes and MICs for ceftazidime, illustrating the correlations among the 23 TR mutants.

The repeat units of the TR mutations that we obtained ranged from 6 to 168 bps in size, and three of them, TRs 12, 13, and 14, were large enough to include the region encoding the entire omega loop (Fig. 1A). Among the 23 TRs, 11 were previously described, particularly with respect to the DNA duplication mechanisms that caused the TRs^10^. Like most ESBLs^19^,^27^, all TR mutants exhibited significantly increased minimum inhibitory concentrations (MICs) for ceftazidime but had decreased MICs for the original substrate, amoxicillin (Fig. 1B). We found a pattern, in which longer duplications generally conferred lower MICs for ceftazidime than did shorter ones. For example, TR14, which has the longest duplication, conferred the lowest MIC for ceftazidime (Fig. 1B). An exception to this pattern is TR4, which conferred a lower MIC than expected for its size (Fig. 1B,C). On the other hand, there were small duplications, TR5 and TR23, that conferred markedly higher MICs for their sizes; notably, TR5 overlaps almost perfectly with the α-helix in the omega loop, while TR23 is just downstream of the α-helix, suggesting that specific structural interventions affecting the α-helix are responsible for the increased MICs for ceftazidime (Fig. 1A–C). When excluding TRs 4, 5, and 23, the correlation between duplication size and MIC improved significantly (Fig. 1C). The MICs for ceftazidime of the mutant enzymes represent the congruent outcomes of the gained ceftazidime hydrolytic activity and the enzyme integrity after structural stabilization. Therefore, the high hydrolytic activity for ceftazidime conferred by the two duplications, TR5 and TR23, may have resulted from an optimal balance between the gained activity and the stability. Consistent with the notion that the α-helix structure may have been specifically affected by these TRs, the two mutations that had the highest MICs among the 29 amino acid substitution mutations in PenL, E166K and L169Q, occurred in the α-helix region^4^. Moreover, two deletion mutations associated with high MICs for ceftazidime, T171del and I173del, were also located just downstream of the α-helix region^20^.

**Figure 2:**
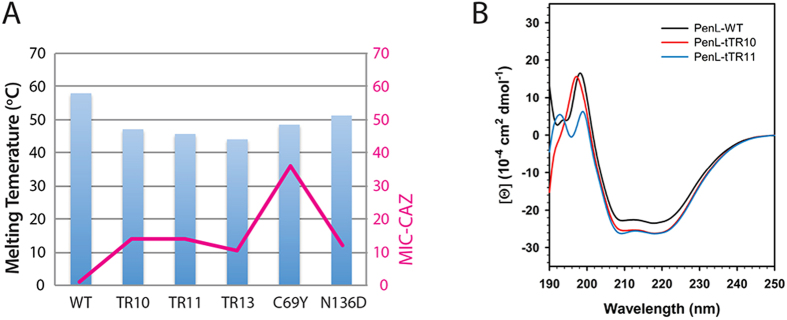
(**A**) Melting temperatures and MICs. Melting temperatures of PenL variants are given along with their MICs for ceftazidime (magenta line). (**B**) Circular dichroism (CD) spectra. CD spectra of PenL variants show the most distinct differences in the far-UV region (190–200 nm).

To test whether longer duplications affect the melting temperature of the enzymes more severely than shorter ones, three duplications of different sizes, TR10, TR11, and TR13, were selected for assays (see Materials and Methods). Thermal shift assays showed that the melting temperature (T_m_) of each enzyme was inversely correlated with the length of the duplication (Fig. 2A). Circular dichroism (CD) spectra of the wild type enzyme and those with TR10 and TR11 mutations showed similar overall profiles, suggesting that major α-helices and β-strands are well maintained regardless of the mutation. Significant changes (decreases in α-helices and β-strands) were mainly localized in the far-UV region (190–200 nm) in both mutant enzymes, and these changes were more prominent in the enzyme with TR11 (a longer duplication) (Fig. 2B). Overall, the data showed that the TR mutations did not result in significant perturbations in the overall enzyme structures, but mainly affected the omega loop region, which likely contributed to the lowered melting temperature of the enzymes (Fig. 2A).

### TR mutant enzymes are specifically active against ceftazidime

To test the TR mutants for their substrate spectrum among β-lactams, in addition to the already known ceftazidime-hydrolyzing capability (Fig. 1), MIC values were measured for various antibiotics, including two penicillins (amoxicillin, penicillin G), two third-generation cephalosporins (ceftotaxime, ceftriaxone), a fourth-generation cephalosporin (cefepime), a monobactam (aztreonam), and a carbapenem (meropenem) (Table S1). A β-lactamase inhibitor, clavulanic acid, was also tested in conjunction with amoxicillin. Unlike that of ceftazidime, the MIC values of the TR mutants for these β-lactams were not higher than those of the wild-type and, in fact, were markedly lower (Table S1). The high susceptibility of wild-type PenL to the inhibitor clavulanic acid was not significantly altered by the TR mutations (Table S1). Together, the data showed that the TR mutations specifically optimized the enzymes for ceftazidime hydrolysis because they were the products from a selection against that particular antibiotic.

### The structures of the wild-type and mutant PenLs

**Figure 3:**
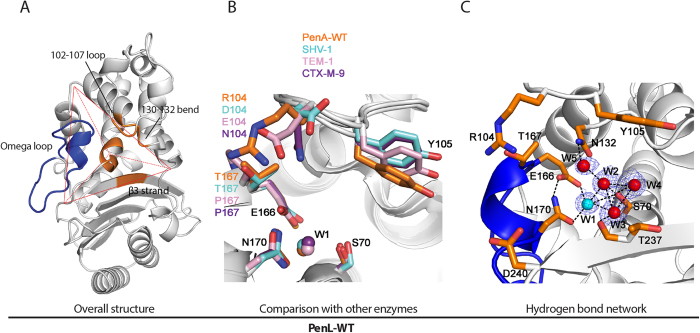
Structure of PenL-WT. (**A**) The overall structure. The active site cavity is depicted with a tetrahedral organization. The omega loop and other conserved regions of the active site are colored blue and orange, respectively. (**B**) Key active site residues of PenL-WT are superimposed with those of other β-lactamases such as SHV-1 (PDB ID: 1SHV), TEM-1 (PDB ID: 1BTL), and CTX-M-9 (PDB ID: 1YLJ). W1: catalytic water. (**C**) Active site of PenL-WT. The hydrogen bond network among the key active site residues and water molecules (red and cyan spheres) with electron density clouds around them is shown. The omega loop is shown as a blue ribbon. Nitrogen and oxygen atoms are colored blue and red, respectively.

In an effort to understand the molecular processes by which structural stress in the omega loop is accommodated and leads to the gains in ceftazidime hydrolytic activity, we analyzed the structures of the wild-type (PenL-WT) and two mutant PenLs (PenL-tTR10 and PenL-tTR11; tTR represents translated TR) to a resolution of 1.5 Å-1.7 Å (Table S2, PDB IDs: 5GL9, 5GLA, and 5GLC, respectively), using the previously solved CTX-M-9 as a search model (PDB ID: 1YLJ). CTX-M-9 shares 55% amino acid identity with PenL-WT and has a low hydrolytic activity against ceftazidime, as does PenL-WT. Similar to other class A β-lactamases, the active site cavity of PenL-WT can be depicted as a tetrahedral organization, where one plane is open for the substrates, while the other three are composed of the omega loop (residues 161–179), structures including the β3 strand (residues 230–240), and the 102–107 loop and the neighboring 130–132 bend (Fig. 3A). These three planes meet at the center, where the N-terminal end of the α2 helix, containing the catalytic Ser70, is located (Fig. 3A). Analysis of the backbone structure of PenL-WT showed high-level structural conservation of the enzyme with homologs in the genus *Burkholderia*, with the calculated root-mean-square deviation (RMSD) values of the C_α_ positions between the enzyme and the PenL homologs from *B. pseudomallei* (PDB ID: 3W4P, 89% amino acid identity) and *B. multivorans*^7^ (PDB ID: 3W4Q, 67% amino acid identity) being 0.36 Å and 0.56 Å, respectively. The RMSD values measured in the other class A β-lactamases, SHV-1 (PDB ID: 1SHV), TEM-1 (PDB ID: 1BTL), and CTX-M-9 (PDB ID: 1YLJ) were in the range of 1.5 to 2.4 Å, also showing high structural similarities to these enzymes. Consistently, the key residues in the active site of PenL-WT were superimposed closely with those in SHV-1, TEM-1, and CTX-M-9 (Fig. 3B).

In addition to these common features, PenL-WT showed two unique features in the active site compared to the homologs: an interaction between Arg104 and Thr167 and an increased number of water molecules in the active site (Fig. 3C). The interaction between Arg104 and Thr167 through a hydrogen bond (2.46 Å) tightened the binding cavity by linking the loop of amino acids 102–107 to the omega loop. Intriguingly, other class A β-lactamases such as SHV-1, TEM-1, and CTX-M-9 do not have such an interaction. The active site of PenL-WT had a fifth water molecule, W5 (Fig. 3C), while class A β-lactamases normally have three to four water molecules, including the catalytic water W1, which has a role in the deacylation reaction of the substrate. All of these water molecules participate in the extensive hydrogen-bond networks that indirectly connect the key active site residues Ser70, Asn132, Glu166, Asn170, and Thr237, which are gathered from all sides of the active site, appropriately positioning them for the catalytic activity (Fig. 3C).

**Figure 4:**
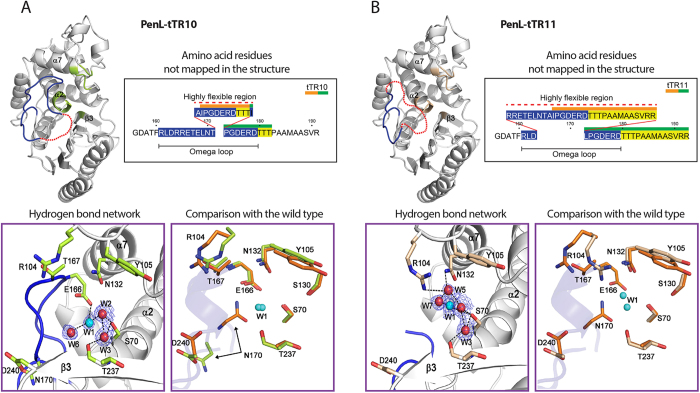
Structures of PenL-tTR mutants. X-ray structures of PenL-tTR10 (**A**) and PenL-tTR11 (**B**) are shown. For each structure of the whole protein, the flexible region not defined in each structure is denoted by a red dotted line drawn based on the approximate length of amino acid residues. Amino acid residues of the tandem repeats (tTRs) are shown in the inset box, where the first and the second repeat units in the tTRs are denoted with orange and green bars, respectively, and the omega loop residues are in blue. The hydrogen bond network among the key active site residues and water molecules (red and cyan spheres) with electron density clouds around them and an overlay of key residues between the mutants (green or tan) and the wild type (orange) are also shown in separate boxes. In these displays, nitrogen and oxygen atoms are colored blue and red, respectively.

**Figure 5:**
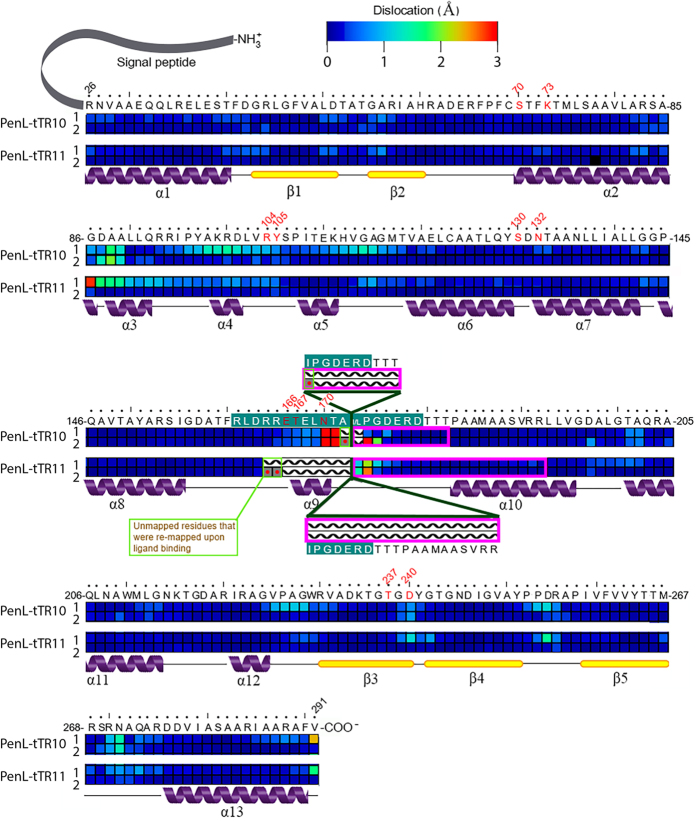
Heat map of α-carbon dislocations of the residues in PenL caused by duplication mutations and substrate binding. The entire enzyme is displayed with the amino acids denoted using the one-letter codes at the top and positions numbered according to Ambler *et al.*^42^. Dislocated residues in PenL-tTR10 and PenL-tTR11, compared to PenL-WT, are displayed separately in the first row. Residues in PenL-tTR10 and PenL-tTR11 dislocated upon substrate binding, compared to PenL-tTR, are displayed separately in the second row. The residues composing the omega loop are shown as white letters on a blue background, and those not mapped in structures due to high flexibility are denoted by a hatched pattern. The repeats are denoted by magenta boxes. Matching secondary structures, α-helices and β-sheets, are displayed below the heat map.

In contrast to the PenL-WT structure, in which every residue could be mapped, PenL-tTR10 and PenL-tTR11 both had a highly disordered region, where the mapping of the residues was not feasible due to high mobility (Fig. 4). The unmapped region in each structure included the entire first repeat unit and the adjoining amino acids in the tTR (Fig. 4). In PenL-tTR10, two additional residues flanking the first unit on either side were not mapped (Fig. 4A). In PenL-tTR11, which has a longer tTR, nine residues in the upstream region of tTR11 were not mapped, in addition to the first repeat unit (Fig. 4B). The remaining tTRs in each structure, composing most of the second repeat unit, replaced the dislocated (unmapped) residues (Fig. 4). In both PenL-tTR10 and PenL-tTR11, it appeared that the α10 helix structure (13 residues: 183–195) downstream of (in the case of PenL-tTR10) or overlapping (in the case of PenL-tTR11) the tTR required the second repeat unit of the tTR, instead of the first unit, to be in place in the protein backbone (Fig. 5). It is reasonable to postulate that the structural perturbations caused by other tTRs are stabilized in a similar way, by extruding the extra residues from the omega loop region. The occurrence of a duplication as long as 56 residues, which included the entire omega loop, demonstrated the high stress-absorbing capacity of the omega loop. Such an adaptive capacity of the omega loop may allow significantly diverse mutations to occur in the loop and to lead to the formation of diverse altered active sites, some of which may have activity against new antibiotics.

### Relaxation of the omega loop results in a widened binding cavity in PenL-tTRs

In the PenL-tTR structures, perturbations in the omega loop resulted in the dislocation of a number of nearby residues, along with the disorganization of the α-helix in the omega loop (Glu166-Asn170), which most notably led to the breakage of a salt bridge that is normally present between Thr167 and Arg104 and tightly packs the active-site cavity (Fig. 4). These disruptions further led to the dislocation of the 102–107 loop; the neighboring α3, α4, α5 helices; and Asp240, which is in the C-terminal section of the β3 strand (Fig. 5).

In addition to the backbone-level dislocations, the side chains of Asn170, Asp240, and Tyr105, which belong to each of the three walls of the active site, respectively, were reoriented, further widening the active site cavity (Fig. 4). In PenL-tTR10, the side chain of Asn170 exhibited a particularly large movement of 9.63 Å, causing it to swing 180° from the active site cavity. Asn170 was found to be further dislocated in PenL-tTR11, resulting in a wider opening in the active site cavity. Furthermore, the side chain of Asp240 was rotated approximately 90°, moving away from the active site by 3.21 Å in PenL-tTR10 and by 2.89 Å in PenL-tTR11. The side chain of Tyr105, which is known to be involved in substrate recognition and the stabilization of the enzyme-substrate complex^28^, was flipped and moved away from the active site by 1.17 Å in tTR10 and by 1.00 Å in tTR11 (Fig. 4). The volume of the active site in PenL-tTR10 was 274.9 Å^3^, as measured with CASTp^29^. This is much larger than that of PenL-WT, which was 182.3 Å^3^, demonstrating the extreme expansion of the active sites in the mutant enzyme. The precise volume of the active site in PenL-tTR11 could not be determined in the same manner because a key residue, Asn170, had swung away from the cavity. However, for the same reason, it is logical to assume that the active site in PenL-tTR11 is larger than that in PenL-tTR10.

The enlargement of the active site was accompanied by reorganization of the hydrogen-bond networks involving water molecules and active site residues (Fig. 4). The original hydrogen bond network inter-connecting Glu166, Asn170, and the catalytic water W1 was disrupted. Instead, a new hydrogen network connecting three water molecules, W1, W2, and W3, and the catalytic Ser70 was formed, maintaining the structural integrity of the active site (Fig. 4). The catalytic water W1 was also connected through a new hydrogen bond with a new water molecule, W6 in PenL-tTR10 (2.99 Å) and W7 in PenL-tTR11 (3.13 Å). Both of these new water molecules were located near the original position of the displaced Asn170, suggesting that water molecules W6 and W7 compensate for the role of the dislocated Asn170, as an anchor for W1 (Fig. 4). In PenL-tTR10, the interaction between W1 and the general base Glu166 remained, although the geometry was altered. This change in the active site was attributed to the low, promiscuous catalytic activity of the mutant enzyme (Table 1). In contrast, the interaction between W1 and Glu166 was lost in PenL-tTR11. In this case, Lys73 may act as an alternative general base. The dispensability of Glu166 in class A β-lactamases has been observed in a number of mutants, in which Glu166 was substituted or deleted, while the enzyme activity was not abolished^30^,^31^.

### Binding of a ceftazidime analog induces reformation of the active site

To determine how a substrate binds to the expanded active site of the PenL-tTRs, we analyzed the structures of PenL-tTR10 and PenL-tTR11 complexed with ceftazidime-like glycylboronic acid (CBA) (PDB IDs: 5GLB and 5GLD, respectively). CBA is a member of the glycylboronates, transition-state analogues of β-lactam antibiotics, which have been used to investigate substrate recognition and catalytic mechanisms in class A β-lactamases^6^,^32^,^33^,^34^. CBA consists of two parts: a glycylboronate moiety that mimicks a transition state intermediate of acylation and a C7β side chain that mimicks the bulky C7β-aminothiazol carboxypropyloxyimino-amide side chain present in ceftazidime^33^.

**Figure 6:**
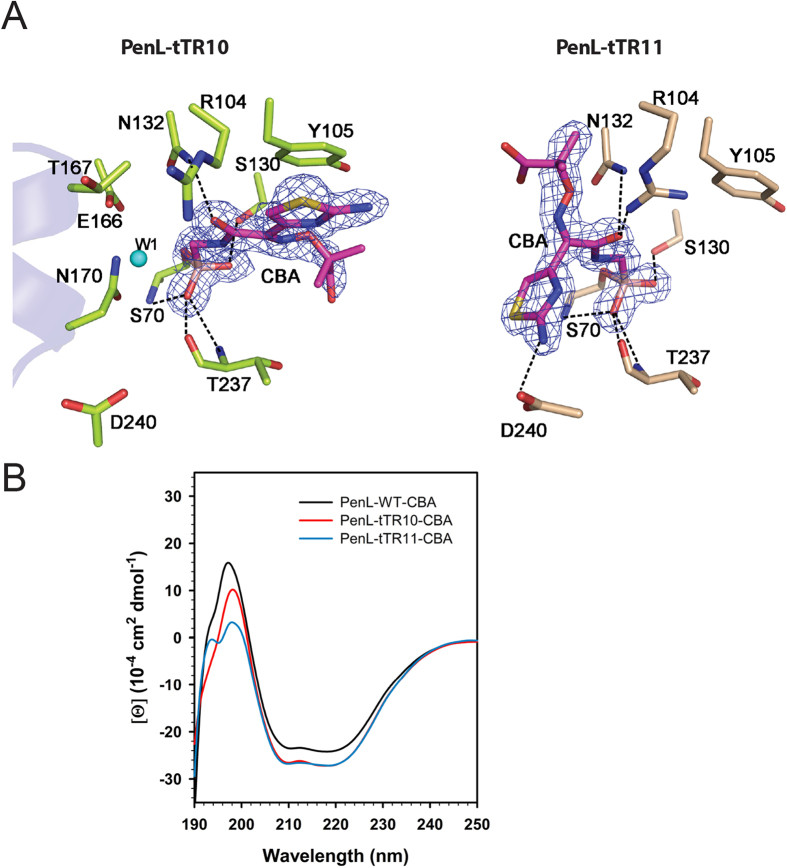
Induced-fit docking of CBA in the active site cavities of PenL-tTR10 and PenL-tTR11. (**A**) The Fo-Fc omit map of CBA is contoured at 2σ. Hydrogen bonds are shown as dotted lines. Nitrogen, oxygen, boron, and sulfur atoms are illustrated in blue, red, pink, and yellow, respectively. (**B**) Circular dichroism (CD) spectra. CD spectra of PenL variants show that the differences between the enzymes in the far-UV region (190–200 nm) are much smaller compared with the spectra without CBA (Fig. 2B).

The binding of CBA reduced the unmapped region by two residues, Ala171 and Ile172, in PenL-tTR10 (Fig. 5). More importantly, the α-helix of the omega loop was restored, consequently reestablishing the catalytic interactions among Glu166, Asn170, and the catalytic water W1 (Fig. 6A). In PenL-tTR11-CBA, the unmapped region was also reduced by two residues, Arg164 and Arg165 (Fig. 5). However, the restoration of the α-helix was not observed in PenL-tTR11-CBA, likely due to the longer tTR11 causing higher disorders in the region (Fig. 6A). CBA was positioned slightly differently in each PenL-tTR (Fig. 6A); in PenL-tTR11, the C7β side chain of CBA presided over the position where the side chains of Glu166 and Asn170 were present in PenL-tTR10-CBA (Fig. 6A). In this position, the bulky C7β side chain appeared to hamper the restoration of the omega-loop α-helix. CBA appeared to be stabilized in the active site of PenL-tTR11 though the interactions of CBA with Asp240 and Arg104. By contrast, the glycylboronate moiety of CBA exhibited a similar binding pattern in both PenL-tTRs, which mimicked the acylation transition state of β-lactam substrates and was similar to the bound conformation to other class A β-lactamases, such as TEM-1, CTX-M-9, and CTX-M-16^6^,^34^ (Fig. S1). The structural information and the enzyme activities against ceftazidime (Fig. 1; Table 1) suggest that the acylation machineries of the PenL-tTRs operated properly, despite the seemingly massive disorganization of the omega loop.

In addition to the interactions occurring with the glycylboronate moiety, those involving the C7β side chain, which further stabilized the PenL-tTR-CBA complexes, were observed. In PenL-tTR10-CBA, the O12 of CBA was hydrogen-bonded to the N_δ2_ of Asn132 and to W5, which was relocated to a position closer to Asn170 (Fig. 6A). In addition, the thiazole ring of CBA had a ring-stacking interaction with the hydroxyphenyl ring of Tyr105 (Fig. 6A). In PenL-tTR11-CBA, the O12 of CBA and N_δ2_ of Asn132 also formed a hydrogen bond, although the distance between the two (3.22 Å) was longer than that in PenL-tTR10-CBA (2.92 Å) (Fig. 6A). Instead of bonding with W5, the O12 of CBA in PenL-tTR11-CBA made a second hydrogen bond (2.31 Å) with the side chain of Arg104, which was kinked and projected into the pocket (Fig. 6A). Moreover, the ring-stacking interaction between CBA and Tyr105 was not observed in PenL-tTR11-CBA, since the thiazole ring of CBA was flipped away from Tyr105 by approximately 120°, unlike that of PenL-tTR10 (Fig. 6A). Instead, CBA complexed with PenL-tTR11 was further stabilized through a hydrogen bond (3.21 Å) between the amino group of its aminothiazole ring and the O_δ1_ of Asp240 (Fig. 6A). Although PenL-tTR10 and PenL-tTR11 exhibited differences from each other in their enzyme-ligand interaction patterns, their binding patterns were largely analogous to those in SHV-1 and CTX-M-9, respectively (Fig. 6A and S1). It is of note that the B factors for CBA in PenL-tTR10-CBA (18.7 Å^2^) and PenL-tTR11-CBA (42.6 Å^2^) were higher than those in SHV-1 (16.1 Å^2^) and CTX-M-9 (10.9 Å^2^), respectively, suggesting more flexible active sites in the PenL-tTRs. Consistent with the structural data, the CD spectra of PenL-tTR10-CBA and PenL-tTR11-CBA showed that much of the altered profiles of the mutant enzymes (Fig. 2B) were recovered upon CBA binding in the far-UV region (190~200 nm), reflecting induced-fit reformation of the active site (Fig. 6B). This recovery of the CD spectrum was more distinct with PenL-tTR10-CBA, which showed a highly-similar profile to that of PenL-WT (Fig. 6B).

Taken together, despite the highly dynamic and relaxed nature of the active sites of the PenL-tTRs, the binding mode of CBA to these proteins deviated little from the common binding paradigm shared among the other class A β-lactamase complexes, demonstrating the functional integrity of the acylation machineries and binding interactions. We conclude that the tTRs in the enzymes resulted in a temporary structural relaxation in the active site cavity, which allowed the access of bulky substrates—such as ceftazidime and CBA—to the cavity, which was re-packed after docking for the subsequent catalytic reactions.

### TR mutations confers an increased affinity to a broad spectrum of β–lactams, which is not necessarily correlated with catalytic activities

The wild-type and three representative mutant enzymes (PenL-WT, PenL-tTR10, PenL-tTR11, and PenL-tTR13) were subjected to kinetic characterizations. While PenL-WT exhibited catalytic activities (k_cat_ and k_cat_/K_m_) with nitrocefin, cefotaxime, penicillin G, and amoxicillin, PenL-tTRs exhibited loss of such activity (Table 1). However, the mutant enzymes, with their expanded, flexible active sites, exhibited a higher affinity (lower K_m_ values) to all β–lactams tested than did PenL-WT, which exhibited particularly low affinity levels to the bulky β-lactams, ceftazidime and cefotaxime (Table 1). Despite their increased affinity to substrates, the mutant enzymes exhibited profoundly low turnover rates (k_cat_ values) with all antibiotics tested (Table 1). The slow turnover rates seemed to greatly override the increased affinity, resulting in overall decrease in the catalytic activity (k_cat_/K_m_ values) to most β-lactams. Intriguingly, the k_cat_ values of PenL-tTRs with ceftazidime were not measurable (Table 1), although MIC values were certainly higher than those of the wild type (Fig. 1B). It has been shown that TEM β-lactamase variants with high affinity to ceftazidime and acylation activity without deacylation can lead to an increased MIC for ceftazidime via the covalent-trapping mechanism^31^. A similar mechanism may underlie the increased resistance to ceftazidime of the mutants with PenL-tTRs. On the other hand, PenL-tTRs were also shown to be more susceptible to catalytic inhibition by the β-lactamase inhibitor clavulanic acid, with IC_50_ values 4 to 5-fold lower than that of the wild type (Table 1).

These results are in accordance with our findings from the structural analyses that PenL-tTRs have flexible and widened active sites, and that their active site residues undergo conformation changes associated with substrate binding, leading to enhanced enzyme-substrate binding interactions. The data also show that PenL-tTRs have catalytic activity for ceftazidime at sub-optimal but physiologically meaningful catalytic efficiencies. This catalytic promiscuity is frequently observed in proteins that have flexible active sites^35^, and is observed in the early variants of ESBLs during their evolutionary development^19^ as the first step before further fine-tuning in the course of evolution.

### Perspectives

The omega loop is characterized by the juxtaposition of the ends of the loop to each other, resembling the shape of a Greek letter Ω^5^. As a surface structure, it is involved in ligand recognition or catalysis in various proteins, such as phospholipase, immunoglobulins, trypsin, and chymotrypsin^5^. The large size range and variable locations of duplications in the omega loop that we characterized in this study demonstrate the extensive adaptive capacity of the omega loop against severe structural perturbations (Fig. 1). It is intriguing to uncover such remarkable flexibility of the omega loop, in that it can re-form by freeing extra residues from its internal structure, while minimizing disruptions in the main structure of the enzyme (Fig. 4).

A common effect resulting from the duplication mutations in the structures of the PenL-tTRs was the disruption of the α-helix in the omega loop structure, which led to a relaxed binding cavity (Fig. 4). However, there were differences in the restorations of the active site conformation during substrate binding in the PenL-tTRs. While PenL-tTR10 reconstituted the original conformation, PenL-tTR11 appeared to exploit the flexibility of the omega loop, allowing large conformational changes in the active site structure^36^. Such active site structures may form an ensemble consisting of a wide range of alternate structural conformers^35^, some of which provide enhanced accessibility of the activated catalytic water to the carbonyl carbon of the acyl intermediate, as suggested for the class C β-lactamase variants^27^,^37^.

From an evolutionary perspective, the omega loop of class A β-lactamases is a newly emerged structural element added to an ancestral penicillin binding protein (PBP), providing a deacylation activity embedded in the omega loop to evolve the PBP into a class A β-lactamase^37^,^38^. The successful evolution of an enzyme may rely on the manipulation of the active site loops while the scaffold and catalytic activity of the ancestral enzyme are maintained^39^. The active site loops can be affected by a variety of mutations, including highly destabilizing ones such as insertions, deletions, and recombinations^39^. With its remarkable adaptability, the omega loop is a hotbed for the evolution of class A β-lactamases, altering their substrate profiles and catalytic efficiencies in response to various selection pressures.

## Methods

### Bacterial strains and cultures

TR mutants of *B. thailandensis* strains were obtained from a large-scale *in vitro* evolution experiment, in which *B. thailandensis* mutants were selected from Luria Bertani (LB) plates containing 3–5 μg/ml of ceftazidime and were identified using sequence analysis, as described previously^4^. All Escherichia coli strains were grown in LB medium, and all B. thailandensis strains were grown in LB medium, Mueller-Hinton (MH) medium or *Agrobacterium* (AB) minimal medium containing 0.25% glucose (ABG) at 37 °C. The concentrations of antibiotics used for *E. coli* were as follows: tetracycline, 10 μg/ml; kanamycin, 50 μg/ml; and ampicillin, 100 μg/ml. For *B. thailandensis*, the concentrations of tetracycline and kanamycin used were 50 μg/ml and 250 μg/ml, respectively.

### Determination of the MIC (minimal inhibitory concentration) values

The MIC values for ceftazidime and amoxicillin were determined using the agar dilution method as described previously^4^. Concentrations of the antibiotics ranged from 0.5 to 32 μg/ml in increments of 2 μg/ml for ceftazidime and from 1 to 64 μg/ml using serial doubling for amoxicillin. MIC values for various other β-lactam antibiotics were measured by E-test following the manufacturer’s instructions (bioMérieux, France) as described previously^4^.

### Production and purification of wild-type and mutant PenLs

The *E. coli* strain DH5α was used as the host for the construction of gene hybrids with protein expression vectors, and *E. coli* BL21 (DE3) was used for protein production. The plasmid pET-28a (+) (Novagen, San Diego, CA, USA) was used for cloning, sequencing, and protein expression. To produce the wild type and mutant PenL enzymes, *E. coli* BL21 cells that were transformed with the appropriate expression constructs were grown at 37 °C in one liter of LB medium containing 50 μg/ml of kanamycin, induced at the mid-exponential phase (A_600_ = 0.6) with 1 mM of isopropyl-β-D thiogalactopyranoside (IPTG), grown for an additional five hours, and harvested by centrifugation (10,000 × g at 4 °C for 20 min). Bacterial pellets were lysed by sonication in buffer containing 20 mM Tris pH 7.5, 500 mM NaCl, 10 mM imidazole, 1 mM phenylmethanesulfonylfluoride, 1 mM dithiothreitol, and DNase I. His-tagged PenL in the lysate was initially purified by Ni-NTA affinity chromatography (Qiagen, Hilden, Germany). The 6xHis tag and residual amino acids from the expression vector were removed by thrombin protease cleavage following dialysis in a thrombin-cleavage buffer (20 mM Tris pH7.5, 100 mM NaCl). The cleaved protein was further purified by Mono S affinity chromatography (GE Healthcare, Piscataway, NJ, USA) and gel filtration on a Hi-Load 16/60 Superdex 200 column (GE Healthcare) in a buffer containing 20 mM Tris-Cl and 50 mM NaCl at pH 7.5. The enzyme purity was assessed using sodium dodecyl sulfate polyacrylamide gel electrophoresis with a 14% separating gel.

### Determination of kinetic parameters

Kinetic assays were carried out following the procedure described by Galleni *et al.*^40^, using the cuvette section of a SpectraMax M2 microplate reader (Molecular Devices, Sunnyvale, CA, USA). Briefly, purified enzymes were diluted with assay buffer (50 mM potassium phosphate buffer (pH 7.0), supplemented with 20 μg/ml of bovine serum albumin (BSA) (Bovogen, Victoria, Australia)). Enzymes in 50 μl of the assay buffer were mixed with 450 μl of a substrate in various concentrations. Reactions were carried out at 25 °C, and the absorbance was analyzed for 90–120 secs at 6-sec intervals using SoftMax Pro V5 software. Steady-state kinetic parameters were calculated by fitting initial rates to the Michaelis-Menten equation (equation 1) using GraFit 7 software (Erithacus Software Ltd., Stains, UK).
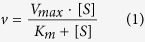
For poor substrates such as ceftazidime and amoxicillin whose kinetic parameters could not be determined using the method described above, competitive assays were performed using nitrocefin as the reporter substrate and the poor substrate as an inhibitor. Reactions were carried out at 25 °C in a final reaction volume of 500 μl containing 50 or 100 μM of nitrocefin, 1.4–176 μM of inhibitor, and 4.5–200 nM of enzyme. Values were calculated from the slope of the line in which *v*_*0*_*/v*_*i*_ values were plotted versus *I* (see equation 2), and K_i_ values of the poor substrates were used as their K_m_ values.
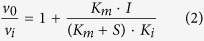
For determination of *k*_*cat*_ values of the poor substrates, *V*_*max*_ values were obtained from the initial rates of the reactions, in which the substrate concentration greatly exceeds the *K*_*m*_ values. Reactions and absorbance data acquisition were carried out as described above.

The IC_50_ for clavulanic acid was determined using competitive assays where 100 μM of nitrocefin was competed against 0–44 μM of clavulanic acid for binding to the enzymes in a 500-μl reaction volume. The IC_50_ was obtained by fitting the reaction rate and the concentration of an inhibitor to the 4-parameter equation of IC_50_ using GraFit 7 software (Erithacus Software Ltd, Stains, UK). Concentrations of the enzymes, substrates and inhibitors used for kinetic assay are summarized in Table 1. All the values listed in Table 1 are shown as the average ± standard deviation of the values obtained from three independent measurements. Molar extinction coefficients and the wavelengths at which absorbance data were acquired are as follows: Nitrocefin, 15000 M^−1^cm^−1^ at 482 nM; Amoxicillin-1100 M^−1^cm^−1^ at 232 nm; Ceftazidime-8660 M^−1^cm^−1^ at 260 nm; Cefotaxime −7500 M^−1^cm^−1^ at 260 nm; Penicillin G −560 M^−1^cm^−1^ at 240 nm.

### X-ray crystallographic data collection, structure determination, and refinement

Purified proteins were concentrated to approximately 7 mg/ml for hanging-drop crystallization trials, for which 1 μl of each protein sample was mixed with 1 μl each of various well solutions. PenL-WT crystals formed in a well solution containing 0.1 M Tris pH 8.5, 0.1 M Li-sulfate, 30% (w/v) PEG 4000, and 15% glycerol within four days at 20 °C. Crystals of PenL-tTR10 and PenL-tTR11 formed in a well solution containing 30% (w/v) polyethylene glycol monomethyl ether 2,000 (PEGMME 2000), 0.1 M K-thiocyanate, and 10% glycerol after one week of incubation at 4 °C.

The crystals were soaked in mother liquor (cryoprotectant) for 30 s and were flash-frozen in liquid nitrogen. X-ray diffraction data were collected at a resolution of 1.5 Å from PenL-WT on the PAL-7A beamline and at a resolution of 1.5 and 1.6 Å from PenL-tTR10 and PenL-tTR11, respectively, on the PAL-5C beamline at the Pohang Accelerator Laboratory (Pohang, Republic of Korea). To obtain crystals of PenL-tTR10 and PenL-tTR11 that were complexed with CBA, protein crystals were soaked with CBA (2 mM) in a cryoprotectant containing 5% dimethylsulfoxide, 30% (w/v) PEGMME 2000, 0.1 M K-thiocyanate, and 10% glycerol overnight at 4 °C, and were then flash-frozen. Data were collected on the PAL-5C beamline, which diffracted the X-rays to 1.6 Å for complexed PenL-tTR10 and 1.7 Å for complexed PenL-tTR11, respectively. All data were processed with HKL-2000 software (HKL Research Inc., Charlottesville, VA, USA).

Unless otherwise stated, model building and refinement were performed with the COOT 9 and REFMAC programs in the Collaborative Computational Project Number 4 suite (CCLRC Daresbury Laboratory, Didcot, UK), and figures were prepared with the PyMOL program ( http://www.pymol.org). Molecular replacement solutions for PenL-WT were obtained with the program MOLREP 10 (correlation coefficient = 79.0%, R-factor = 34.9%) and using CTX-M-9 (PDB code: 1YLJ) as a search model with the water and ions removed. The refined PenL-WT PDB was used as a search model for further molecular replacements for PenL-tTR10, PenL-tTR11, PenL-tTR10-CBA, and PenL-tTR11-CBA, resulting in a correlation coefficient of 92.4% and R-factor of 24.4% for PenL-tTR10, a correlation coefficient of 91.6% and R-factor of 24.2% for PenL-tTR10-CBA, a correlation coefficient of 92.2% and R-factor of 24.4% for PenL-tTR11, and a correlation coefficient of 91.9% and R-factor of 24.7% for PenL-tTR11-CBA. In particular, the CBA PDB file was created using the PHENIX program LIGANDFIT^41^ and was fitted into a Fo-Fc density that was consistent with the ligand during the building of the PenL-tTR10-CBA and PenL-tTR11-CBA models in COOT. The refinements for all of the models that could be expected at those resolutions converged to reasonable values (Table S2).

### Protein thermal shift assay

Melting curves of PenL-WT and the PenL-tTRs were obtained using a Protein Thermal Shift^TM^ kit (Applied Biosystems, Foster City, CA, USA) following the manufacturer’s instructions. Briefly, purified proteins were mixed with ROX, a fluorescent dye, to a final concentration of 15 μM and were subjected to a melting curve experiment on a 7500 Real-Time PCR machine (Applied Biosystems). The protein melting reaction was carried out in a total volume of 20 μl and consisted of two steps: (1) temperature, 25 °C; time, 2 min; ramp rate, 100% and (2) temperature, 99 °C; time, 2 min; ramp rate, 1%. The obtained melting curve data from the 7500 software were imported into the Protein Thermal Shift^TM^ software (Applied Biosystems) and further analyzed to calculate the Boltzmann melting temperature, T_m,_ which is the reflection point in the original sigmoidal melting curve. The average of three repeated experiments was reported.

### Circular dichroism spectra assay

Circular dichroism (CD) spectra were measured with a Jasco J-810 spectropolarimeter with a Peltier temperature-controlled cuvette holder. The CD spectra of the enzyme samples (25 μM, ~0.7 mg/ml) in a buffer (20 mM Tris-Cl, pH 7.5, 50 mM NaCl) were measured using a cuvette with a 0.1-cm path length, in the far-UV region (190–250 nm). Baselines were determined using the buffer solution from the final gel-filtration step of protein purification. Triplicate scans were run for each enzyme at 0.1 nm intervals with a 1 nm bandwidth with and without CBA. For CD scans of the PenL-CBA complexes, concentrated CBA (100 μM) prepared in the same buffer was diluted to 50 μM, either in protein solution or buffer, and incubated at room temperature for 1 hr prior to the measurement. The trace for PenL-CBA was corrected by subtracting the spectral values of the solution containing only 50 μM of CBA. Data are presented as the average of the three CD spectra, after smoothing using Spectra Manager^TM^ (ver 2.8).

## Additional Information

**How to cite this article**: Yi, H. *et al.* High adaptability of the omega loop underlies the substrate-spectrum-extension evolution of a class A β-lactamase, PenL. *Sci. Rep.* **6**, 36527; doi: 10.1038/srep36527 (2016).

**Publisher’s note**: Springer Nature remains neutral with regard to jurisdictional claims in published maps and institutional affiliations.

## Supplementary Material

Supplementary Information

## Figures and Tables

**Table 1:** Kinetic parameters of PenL variants.

		K_m_ (μM)	k_cat_ (s^−1^)	k_cat_/K_m_ (μM^−1^s^−1^)	IC_50_ (μM)
NFN	WT	29.4 ± 2.4	325 ± 44	11	
	tTR10	8.9 ± 0.5	1.6 ± 0.12	0.18	
	tTR11	6.1 ± 0.3	1.06 ± 0.14	0.17	
	tTR13	2.8 ± 0.2	0.33 ± 0.03	0.12	
CAZ	WT	NM	NM^d^	—	
	tTR10	9.7 ± 0.3^b^	NM	—	
	tTR11	23.9 ± 12^b^	NM	—	
	tTR13	2.8 ± 0.7^b^	NM	—	
CTX	WT	452.7 ± 64.8	1067 ± 255.5	2.4	
	tTR10	1.6 ± 0	0.069 ± 0.0089	0.0431	
	tTR11	6.8 ± 7.2	0.054 ± 0.0177	0.0079	
	tTR13	ND	ND	ND	
PGL	WT	119.3 ± 13.3	243.7 ± 27.4	2	
	tTR10	4.5 ± 0.2^b^	NM	—	
	tTR11	4.3 ± 0.7^b^	NM	—	
	tTR13	ND	ND	ND	
AMX	WT	162 ± 64^b^	83.5 ± 10.8^c^	0.5	
	tTR10	4.5 ± 0.2^b^	NM	—	
	tTR11	11.2 ± 0.3^b^	NM	—	
	tTR13	1 ± 0.3^b^	NM	—	
CLA	WT				1.92 ± 0.8
	tTR10				0.35 ± 0.023
	tTR11				0.42 ± 0.127
	tTR13				ND

^a^Substrate and inhibitor: NFN, nitrocefin; CAZ, ceftazidime; CTX, cefotaxime; PGL, penicillin G; AMX, amoxicillin; CLA, clavulanic acid.

^b^K_i_ was used as K_m._

^c^k_cat_ was calculated using V_max_, which was determined as the initial reaction rate at which substrate concentration exceeded K_m_ values by more than 10-fold.

^d^ND: not determined, NM: not measurable.
