# Supplementary material for: High adaptability of the omega loop underlies the substrate-spectrum-extension evolution of a class A β-lactamase, PenL
Source: Sci Rep. 2016 Nov 9;6:36527. doi: 10.1038/srep36527 (PMC5101513; doi:10.1038/srep36527)
Supplement: Supplementary Information [file srep36527-s1.pdf]

Supplementary file for:

High adaptability of the omega loop underlies the substrate-spectrum-extension evolution of a class A β-lactamase, PenL

Hyojeong Yi[1†], Jin Myung Choi[2†], Junghyun Hwang[1], Fabio Prati[3], Thinh-Phat Cao[2], Sung Haeng Lee[2*], and Heenam Stanley Kim[1*]

[1]Department of Biomedical Sciences, Korea University, Anam-Dong, Seongbuk-Gu, Seoul 136-705, Korea; [2]Department of Cellular and Molecular Medicine, Chosun University School of Medicine, Gwangju 501-759, Korea; [3]Department of Life Sciences, University of Modena, Modena 41125, Italy

This file includes:
Figure S1 and Tables S1 and S2

Figure S1. Comparison of CBA binding between PenL-tTRs and other class A β-lactamases. A. Comparison of the CBA binding mode in PenA-tTR10 (wheat) and SHV-1 (aquamarine, PDB ID: 3MKE). B. Comparison of the CBA binding mode in PenL-tTR11 (yellow) and CTX-M-9 (violet, PDB ID: 1YLY). Hydrogen bonding or ring-stacking interactions are shown as dotted lines. CBAs bound to TR mutants are shown in lime, whereas those bound to SHV-1 or CTX-M-9 are shown in magenta. Nitrogen and oxygen atoms are colored blue and red, respectively.

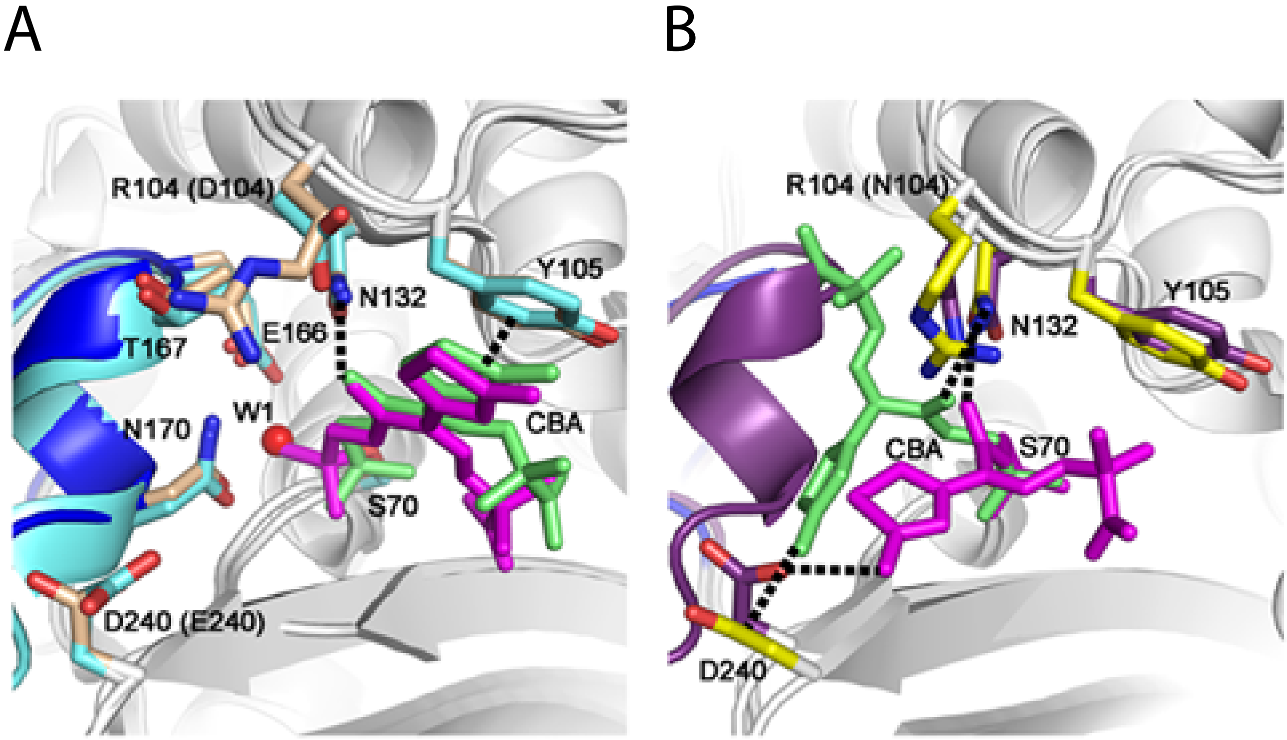

Table S1. MICs for various β-lactams of PenL variants[a]

Strain	PGL	AMX	AMC	CTX	CRX	CEF	AZT	MRP
WT	>32[b]	48	6	8	26.7	14	192	0.5
TR1	32	6	1.5	1.5	1.8	4	2.3	0.5
TR2	16	4.3	1.3	2.2	2.2	4.3	2.3	0.7
TR3	9	4.3	1.3	1.7	2.5	3.7	1.8	0.5
TR4	19	3.7	1.5	1	1.8	3.3	1.5	0.5
TR5	26	6	1.7	2.7	3.7	6	2.7	0.5
TR6	20	4.7	1.7	2.2	2.7	6	2	0.7
TR7	22	5	1.5	1.8	2.7	5.7	1.8	0.5
TR8	18	6	1.5	1.5	2.7	4.3	2.7	0.5
TR9	20	5.3	1.5	1.5	2.7	5.3	2.2	0.5
TR10	7	5.3	1.3	1.5	2.3	4.3	1.8	0.5
TR11	7	4.7	1.3	1.5	2.2	4.3	2.2	0.5
TR12	18	5	1.3	1.3	2.2	4.3	1.7	0.5
TR13	16	4.7	1.5	1.5	2.3	4.7	1.8	0.6
TR14	14	5.3	1.7	1.3	1.8	3.7	2.2	0.5
TR15	12	4.3	1.5	1.7	2.2	4.3	1.8	0.6
TR16	12	4	1.3	1.8	2.7	5.3	6.2	0.8
TR17	20	5.3	1.5	2.2	3	6	2.7	0.7
TR18	22	6	1.3	2.2	2.3	4.7	3	0.6
TR19	12	3.7	1.5	3.3	4.3	6.7	2.3	0.5
TR20	>32	6	1.7	1.8	2.7	5.3	2.7	0.5
TR21	24	5.3	1.7	2.7	3.3	5.3	3	0.5
TR22	>32	7.3	1.7	2.7	3.7	6.7	3.7	0.5
TR23	>32	10	1.8	4.7	5.3	7.3	3	0.6

[a]Substrate and inhibitor : PGL, penicillin G; AMX, amoxicillin; AMX, amoxicillin/clavulanic acid; CTX, cefotaxime; CRX, ceftriaxone; CEF, cefepime; AZT, aztreonam; MRP, meropenem.
[b]The unit of MIC is μg/ml.

Table S2. Statistics for data collection and refinement of PenL proteins

	PenL				
**Parameter**	**WT**	**TR10**	**TR10-CBA**	**TR11**	**TR11-CBA**
**PDB code**	5GL9	5GLA	5GLB	5GLC	5GLD
**Data collection**					
Wavelength (Å)	0.97934	0.97951	0.97951	0.97954	1.00000
Resolution (Å)	50-1.5 (1.53-1.5)	50-1.5 (1.53-1.5)	50-1.6 (1.63-1.6)	50-1.6 (1.63-1.6)	50-1.7 (1.73-1.7)
Space group	$P2_12_12$	$P2_1$	$P2_12_12$	$P2_12_12$	$P2_12_12$
Unit cell dimension					
a, b, c (Å)	75.93, 81.64 36.83	37.03, 92.55 68.87	70.06, 92.88 34.99	69.49, 97.24 34.00	70.27, 97.45 34.22
α, β, γ (°)	α=β=γ=90°	α=γ=90°, β=92.61°	α=β=γ=90°	α=β=γ=90°	α=β=γ=90°
Total reflections	535224	363038	286864	442064	205607
Unique reflections	36567 (1757)	69882 (3484)	30982(1487)	31195(1532)	26287 (1274)
R_{merge}† (%)	5.4 (12.5)	6.1 (20.7)	7.2 (31.5)	7.4 (33.2)	9.3 (35.1)
Completeness (%)	98.7 (97.8)	99.9 (100)	100 (100)	99.9 (100)	99.9 (100)
Redundancy	14.4 (14.5)	5.2 (5.1)	9.3 (9.4)	14.2 (14.2)	7.8 (7.9)
Average I/σ (I)	18.4 (31.1)	18.4 (9.7)	12.8(9.7)	15.5 (12.3)	16.0 (9.5)
**Refinement**					
R_{work} / R_{free} (%)	16.3/19.0	17.0/19.6	16.4/20.0	16.0/18.4	15.9/20.0
Protein residues	268	533	268	255	257
Waters	342	569	254	267	277
CBA			1		1
RMSD					
Angle (°)	1.057	1.073	1.195	1.124	1.124
Length (Å)	0.006	0.006	0.007	0.007	0.006
Average B factors ($Å^2$)	20.0	14.0	7.0	16.0	27.0
Ramachandran plot					
Favored (%)	98.1	96.7	98.1	97.6	98.0
Allowed (%)	1.9	2.90	1.9	2.0	2.0
Outliers (%)	0	0.4	0	0.4	0

Values in parentheses correspond to highest resolution shell.

†R-merge=$\Sigma_{hkl}\Sigma_i |I_i(hkl)-\langle I(hkl)\rangle| / \Sigma_{hkl}\Sigma_i I_i(hkl)$, where $I_i(hkl)$ is the observed intensity and $\langle I(hkl)\rangle$ is the average intensity of symmetry-related observations.
